# Montelukast Use and Patterns of Ambulatory Care among Asian versus Non-Asian Adult Patients with Asthma and/or Allergic Rhinitis in the United States

**DOI:** 10.36469/9835

**Published:** 2015-12-16

**Authors:** Joseph Vasey, Shalini Bagga, Huan Huang, Tongsheng Wang, David Thompson

**Affiliations:** 1 Practice Fusion, San Francisco, CA; 2 Merck & Co., Inc., Kenilworth, NJ; 3 Quintiles, Cambridge, MA; 4 Bristol-Myers Squibb, Hopewell, NJ

**Keywords:** asian, ambulatory care, montelukast, allergic rhinitis (ar), asthma

## Abstract

**Background:** Asthma and allergic rhinitis (AR) are inflammatory conditions that are similar in pathophysiology. Mild-to-moderate persistent asthma has been widely treated with inhaled corticosteroids, while allergic rhinitis is commonly treated with antihistamines, nasal corticosteroids, anticholinergics, and other allergy specific medications. The introduction of montelukast, a leukotriene receptor antagonist, has opened a treatment pathway that is common to both conditions. Previous real world studies of montelukast (Singulair®) relative to other medications have not investigated the role of race in the management of asthma and AR, specifically as relates to differences among Asian versus non-Asian patients.

**Objective:** To contrast montelukast use and patterns of ambulatory care for adult Asian versus non-Asian patients in the United States with asthma and/or AR.

**Methods:** Data for adult asthma and AR patients were extracted from a national electronic medical records database for the years 2006-2014. Patients were classified into condition cohort (Asthma-Only, AR-Only, Asthma & AR), and treatment condition (monotherapy or combination therapy, with or without montelukast for Asthma and Asthma & AR cohorts, usual care with or without montelukast for AR-Only) and stratified by race (Asian vs. non-Asian).

**Results:** Overall patterns of use of montelukast were similar for Asian and non-Asian patients, but Asians were more likely to receive it as part of a combination therapy regimen. Changes in treatment regimen followed similar patterns for both groups. Asian patients with both asthma and AR were found to have lower service utilization rates if their therapy included montelukast, whereas for non-Asians there was no significant difference between regimens with or without montelukast.

**Conclusion:** Differences in montelukast use and outcomes of care exist between Asian and non-Asian patients in the United States. Future research should explore the reasons for these differences and whether they can be replicated in non-US settings.

## BACKGROUND

Asthma and allergic rhinitis (AR) are two of the most common problems affecting the respiratory system.[1] Although these conditions are distinct, there are epidemiological and clinical data suggesting a comorbid relationship between asthma and AR.[2] Both are chronic inflammatory conditions, with an overlapping prevalence and economic burden. They have similar pathophysiology and therefore share some aspects of patient management.[1] It has been reported that 40% of AR patients have asthma, and 30-80% of asthmatic patients have AR.[1] In addition, in the mid-1990s total direct costs of AR in the US were between US$1.2 and US$3.4 billion, compared with US$6.1 billion for asthma.[3] In less than five years the annual cost of asthma in the US was estimated to be approximately $12.7 billion.[1,3] AR is responsible for approximately 811 000 missed workdays, 824 000 missed school days, and 4 230 000 reduced activity days.[4] Children with both asthma and AR have higher pharmacy, physician, and hospital service costs than children with asthma alone.[5]

For many years, mild to moderate persistent asthma was treated almost exclusively with inhaled corticosteroids (ICS). More recently, leukotriene receptor antagonists (LTRAs) have emerged as an alternative asthma therapy with a different mechanism of action.[6] According to the Global Initiative for Asthma (GINA) guidelines, montelukast (Singulair; Merck) is the recommended alternative monotherapy to low-dose ICS, especially in a step-down strategy.[7] It is also used as an add-on treatment to ICS plus long-acting beta-agonist (LABA) combination, in order to improve control and reduce the dose of ICS.[7] It has proven to be particularly effective in exercise-induced asthma and in asthma associated with AR.[7] Montelukast blocks the action of leukotrienes, inflammatory agents involved in both asthma and AR, making it the only treatment indicated for both conditions.

In the United States the Asian population is growing faster than any other race group. The total population has grown from 281.4 million in year 2000 to 308.7 million in year 2010 (a 9.7% increase), while the Asian population has increased from 10.2 million to 14.7 million (an increase of 44.1%).[8] Available research estimates the prevalence of asthma among Asian patients in the United States to range from 11-24%, with variations attributable to generational status, degree of acculturation, US born versus immigrant, and socio-economic status.[9]

It has been reported by the US Department of Health and Human Services that Asian Americans generally have lower rates of asthma than the white population, however their death rate is higher.[10] Yet, in spite of the significant growth of the Asian subpopulation and the higher death rate, there are no published studies that directly compare any asthma treatment effects between Asian and non-Asian patients in the United States. It is challenging to compare the outcomes reported in the Asian population to those reported in the non-Asian population based on different studies, due to the inconsistency in patient population, outcome definition, and follow-up period.[11-13]

There are a few real-world studies of montelukast versus other treatments based on claims data,[12,14,15] but none of these studies contrasted patterns of care for Asian versus non-Asian patients. A potential reason for the lack of information about Asian asthma patients is the past reliance on claims data, which often contains incomplete or missing data on enrollee race. Electronic medical records (EMR) offer an alternative data source that provides information on ambulatory care, medications, and patient characteristics, including race. The objective of this study was to use EMR data to contrast montelukast use and patterns of ambulatory care for adult Asian and non-Asian patients in the United States with asthma and/or AR.

## METHODS

### Data Source

The data source for this study was a nationwide ambulatory care EMR database containing data on more than 39 million patients overall and over 17 million active patients as of March 2014. The EMR includes more than 600 member groups with 39 000+ providers covering 49 States and the District of Colombia. Its providers represent a variety of practice types, ranging from sole practitioners to community clinics, academic medical centers, and large integrated delivery networks. The majority of providers are primary care physicians.

Data are entered into the system by health care providers in real time. The data represent each patient's medical record and provide patient-level information including patient demographic (age, gender, race) and clinical characteristics (BMI, height, weight), patient encounter dates and details, prescribing physician specialty, laboratory values, comorbid medical conditions, as well as prescription and over-the-counter (OTC) medications.

### Cohort Selection & Definition of Treatment Groups

Adult patients (18 year of age or older) with a diagnosis of asthma and/or AR were identified from the EMR database within the study period January 2006 through March 2014. To be a candidate for the Asthma-Only cohort, patients were required to have at least two clinical activities (i.e., a visit, test, prescription or refill order, education, consultation) with a diagnosis of asthma (ICD-9-CM 493.xx) during the study period. To be a candidate for the AR-Only cohort, patients were required to have at least two clinical activities with a diagnosis of allergic rhinitis (ICD-9-CM 477.xx) during the study period. All patients who had at least two clinical activities for both diagnoses were candidates for inclusion in the Asthma & AR cohort.

Some exclusions were made to ensure our analyses were focused on patients being treated solely for asthma and/or AR, consistent with other published studies.[14-16] Patients with a concurrent diagnosis of chronic obstructive pulmonary disease (COPD, ICD-9-CM code 491.xx, 492.xx, 496.xx), chronic bronchitis (ICD-9-CM 491.xx), emphysema (ICD-9-CM 492.xx), bronchopulmonary dysplasia (ICD-9-CM 770.7x), or cystic fibrosis (ICD-9-CM 277.0x) at any time during the study period were excluded. Due to the analytic focus contrasting Asian to non-Asian patients, those with undetermined or unreported race were excluded as well.

The algorithm for treatment assignment is illustrated in Figure 1. For the Asthma-Only and Asthma & AR cohorts, patients were classified into one of four treatment groups based on observed patterns of prescription order(s): montelukast monotherapy, ICS monotherapy, montelukast combination therapy (ICS+montelukast or ICS+LABA+montelukast), or ICS combination therapy (ICS+LABA). (During most of the period covered by this study montelukast was available only under the brand name Singulair; for consistency, in prescription records from 2012 onward we required specification of the brand rather than generic form of the drug.) The date of the first-observed prescription order was designated the index date and additional prescriptions (if any) were assessed during the 60-day period following the index date to distinguish between mono versus combination therapy, consistent with other research.[14] Patients who received montelukast with their index prescription and had no other prescription orders during the 60-day period were placed in the montelukast monotherapy group; those who had orders for other asthma medications were placed in the montelukast combination therapy group. Similarly, patients who received ICS as their index prescription and had no other prescription orders during the 60-day period were placed in the ICS monotherapy group; those who had orders for other asthma medications were placed in the ICS combination therapy group.

**Figure 1.** Treatment Group Assignment

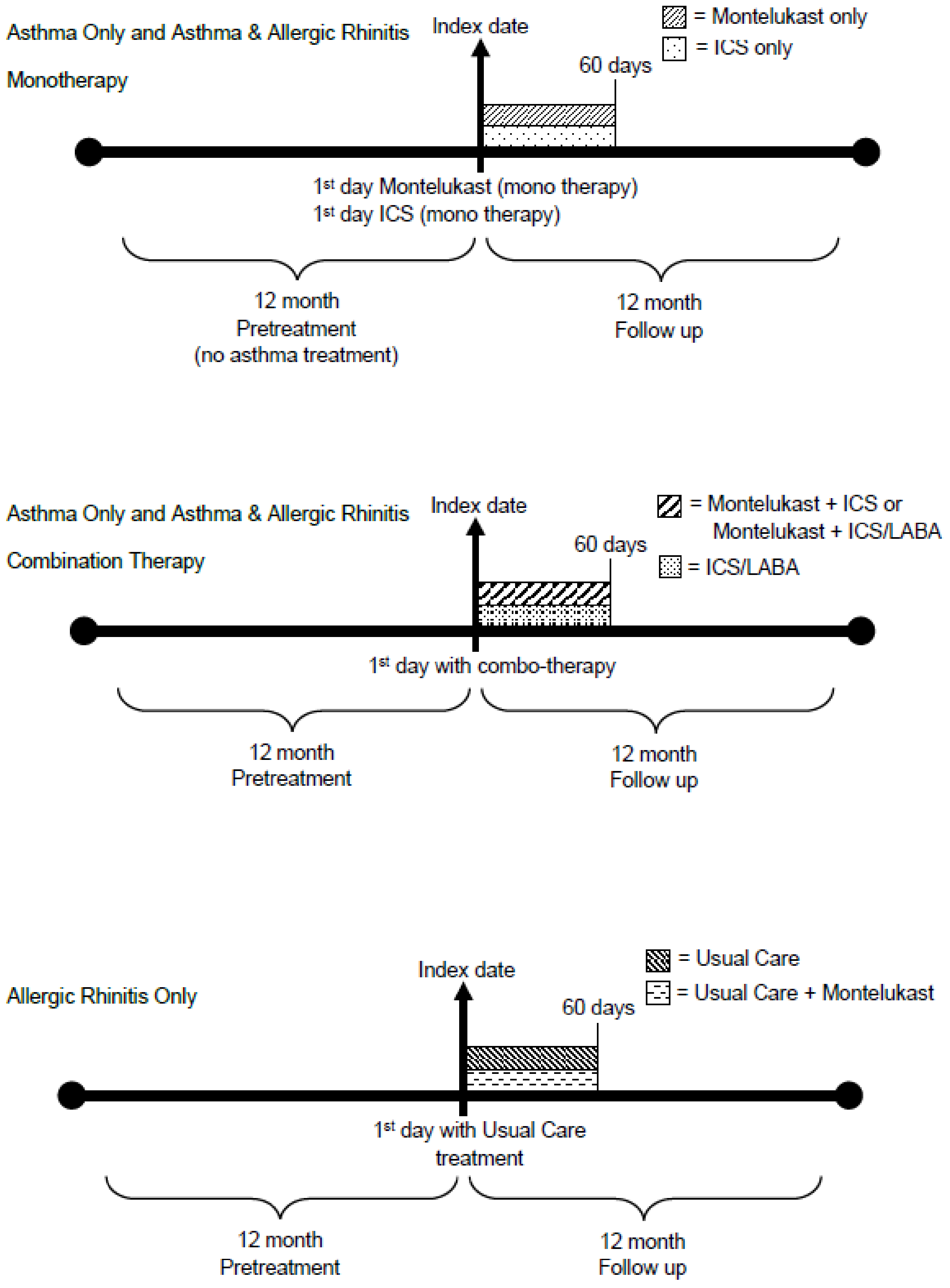

ICS: inhaled corticosteroid

For the AR-Only cohort, patients were classified into one of two treatment groups, usual care or usual care plus montelukast. Patients who received antihistamines or antihistamine combinations, nasal corticosteroids, or other medications (anticholinergics, mast cell stabilizers, sympathomimetic agents, or alpha agonists) and no prescription for montelukast during the 60-day period were placed in the usual care group. Individuals with similar prescriptions plus montelukast were placed in the montelukast plus usual care group.

### Study Measures

Measures of patterns of care were limited to activities and actions recorded in the ambulatory record. Physician office visits during follow-up were assessed. Changes (switching or augmentation) of the index treatment were assessed for the Asthma-only and Asthma & AR cohorts. Switching was defined as the presence of an order for a non-index medication without a refill of the index medication in the 90 days following the end of the supply of the previous order for the index prescription.[17] Augmentation was defined as the addition of a non-index prescription in conjunction with refills of the index medication within 60 days of the addition of the new treatment.[14] For patients who switched or had an add-on to the index treatment, descriptive analyses were conducted on what treatment patients switched to or added.

For the AR-Only cohort, the percentage of patients who received orders for each of several types of AR medication (antihistamine, nasal corticosteroid, montelukast, other) during follow-up was evaluated. Orders for prescription corticosteroids were assessed for all patients.

### Statistical Analyses

Pretreatment patient demographic (age, gender) and clinical characteristics (BMI, smoking status, asthma related comorbidities) were evaluated for all patients. Comorbidities of interest included hypertension, sleep disorder, obesity, sinusitis, tonsillitis, rhinorrhea, acute upper respiratory infection, conjunctivitis, chronic otitis media, pharyngitis, and nasal polyposis. The relevance of these comorbidities to asthma has been established in prior research.[14-16,18-20] Asthma or AR-related medication use during the pretreatment period was evaluated for all patients. The statistical significance of differences by treatment group was evaluated using Chi-square tests, with significance set at the $p<0.05$ level.

For utilization measures during the follow-up period, comparisons were carried out between patients on a regimen that included montelukast versus a comparable one that did not. For the Asthma-Only cohort and the Asthma & AR cohort, patients receiving montelukast as monotherapy were compared with patients receiving ICS monotherapy, and patients receiving montelukast as combination therapy were compared with patients receiving an ICS combination. For the AR-Only cohort, patients receiving usual care were compared with those receiving usual care plus montelukast. All analyses were stratified by Asian versus non-Asian race. The statistical significance of differences by treatment group was assessed using a Chi-square test for categorical variables, and Wilcoxon rank sum test or t-test for continuous variables depending on the distribution of data. Throughout, statistical significance was defined as $p<0.05$.

The effect of including montelukast on the number of office visits was assessed by estimating an adjusted mean number of visits in each treatment regimen (monotherapy or combination therapy for Asthma-Only and Asthma & AR cohorts; usual care for AR-Only). This was calculated through the use of covariate adjusted negative binomial regression, a technique appropriate for count data. Covariates included gender, age group, obesity status, smoking status, and comorbidities.

## RESULTS

### Sample Selection & Patient Attrition

Approximately 3.4 million patients in the EMR database had diagnoses of asthma and/or AR in the period 2006-2014, including 1 million Asthma-Only, 1.8 million with AR-Only, and over 500 000 Asthma & AR patients. Figure 2 illustrates attrition from each cohort during the sample selection process—including exclusions for age less than 18 years, unknown race, and selected pulmonary comorbidities—which resulted in final cohorts of 93 590 Asthma-Only patients, 431 769 AR-Only patients, and 76 218 Asthma & AR patients. Figure 3 depicts the assignment of patients to treatment groups within each cohort. Patients with a prescription for montelukast, either alone or in combination with other therapies, made up 14% of the Asthma-Only cohort, 7% of the AR-Only cohort, and 22% of the Asthma & AR cohort. Most Asthma-Only and Asthma & AR patients with a montelukast combination therapy had prescriptions for ICS and montelukast (83.7% of Asians with Asthma-only; 82.1% of non-Asians with Asthma-only; 87.0% of Asians with Asthma & AR, and 86.0% of non-Asians with Asthma & AR).

### Patient Characteristics

Table 1 presents patient demographic and clinical characteristics by treatment group within each cohort. Overall, 3.2% of the sample (18 979) were Asian, 79.5% were white, and 11.9% were black. Across cohorts and treatment groups Asian patients accounted for 1.7 to 3.6% of the sample. Female patients were predominant (67.8%). The average age was 48.7 years. BMI, available for 68.7% of the sample, indicated that more than half of patients (55.5%) with reported BMI were not overweight or obese. Smoking status was available for approximately one-third of the sample. Among those where it could be determined, 60.9% had never smoked, 22.2% were former smokers, and 16.9% were current smokers. The most commonly reported comorbidities were hypertension (28.3%), sleep disorders (10.7%), and sinusitis (10.3%).

### Patterns of Medication Use

Differences were observed in the use of montelukast between Asian and non-Asian patients. In both the Asthma-Only and Asthma & AR cohorts, the overall use of montelukast was similar, but Asians were more likely than non-Asians to receive the product as combination therapy (6.3% vs. 5.7% among Asthma-Only patients and 10.8% vs. 10.0% among Asthma & AR patients) as opposed to monotherapy (6.7% vs. 8.3% and 9.6% vs. 11.9% respectively). In the AR-Only cohort, montelukast was less used among Asians than among non-Asians (4.1% vs. 6.6%).

Patients on a monotherapy were significantly less likely to experience a change in that therapy than those on a combination therapy (Table 2). Between 68-80% of patients on monotherapy received no additional medications during the year following treatment initiation. Combination therapies were significantly more likely than monotherapies to be changed during the follow-up period, regardless of patient race or diagnosis. Augmentation was more common among Montelukast monotherapy than ICS monotherapy patients, regardless of race or diagnosis. Augmentation occurred in nearly half or more of patients' initial combination therapies. Prescriptions for Asian and non-Asian asthma patients had similar rates of augmentation regardless of the type of combination therapies. For asthma & AR patients, ICS combination therapy was more likely to be augmented than montelukast combination therapy, regardless of race – for Asians, only 47% of Montelukast combination therapy patients were augmented compared to 55% for ICS combination; for non-Asians, similar comparison was 48% vs. 57%.

**Figure 2.** Attrition of Patients during the Sample Selection Process

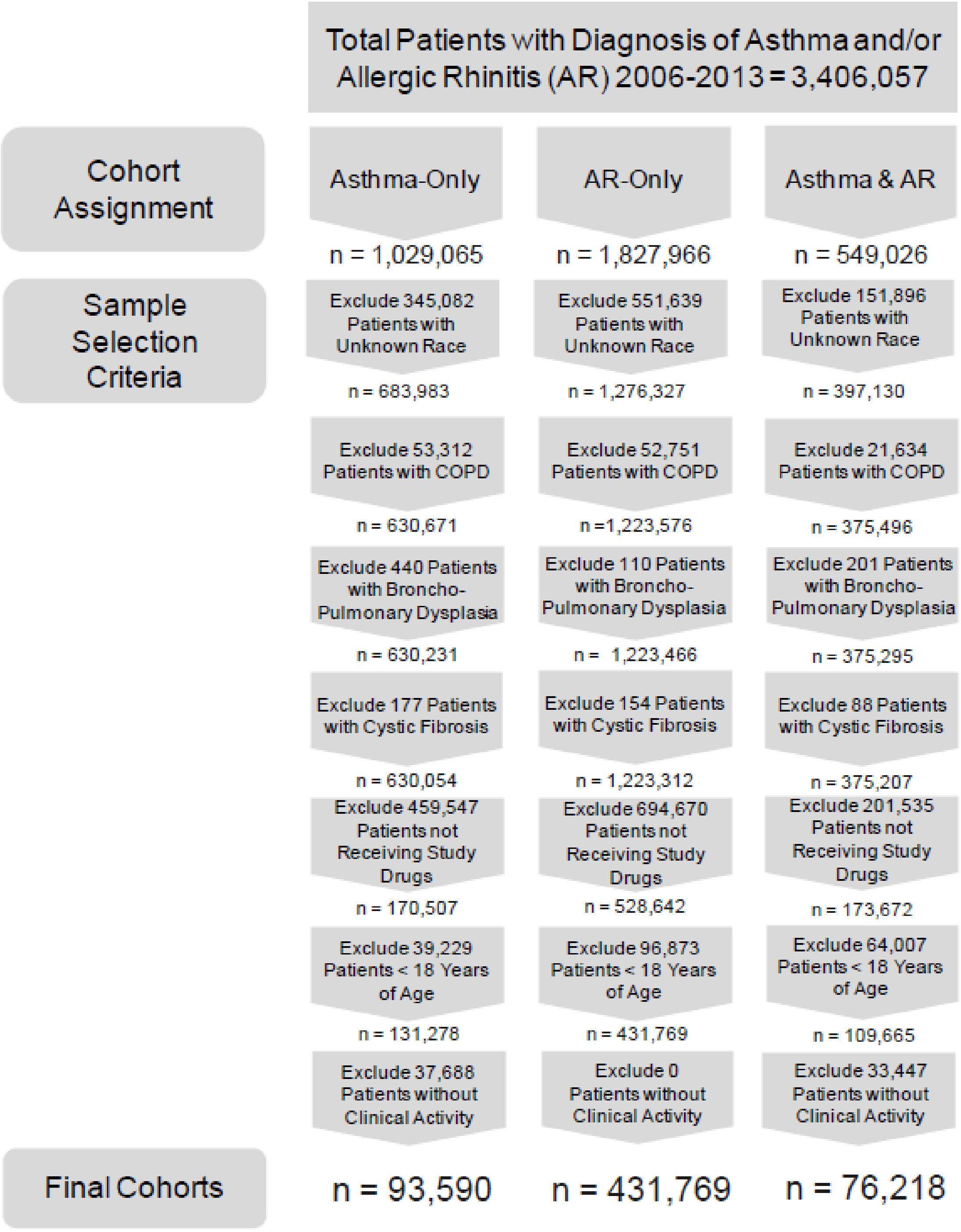

**Figure 3.** Flow of Patients to Treatment Group Assignment

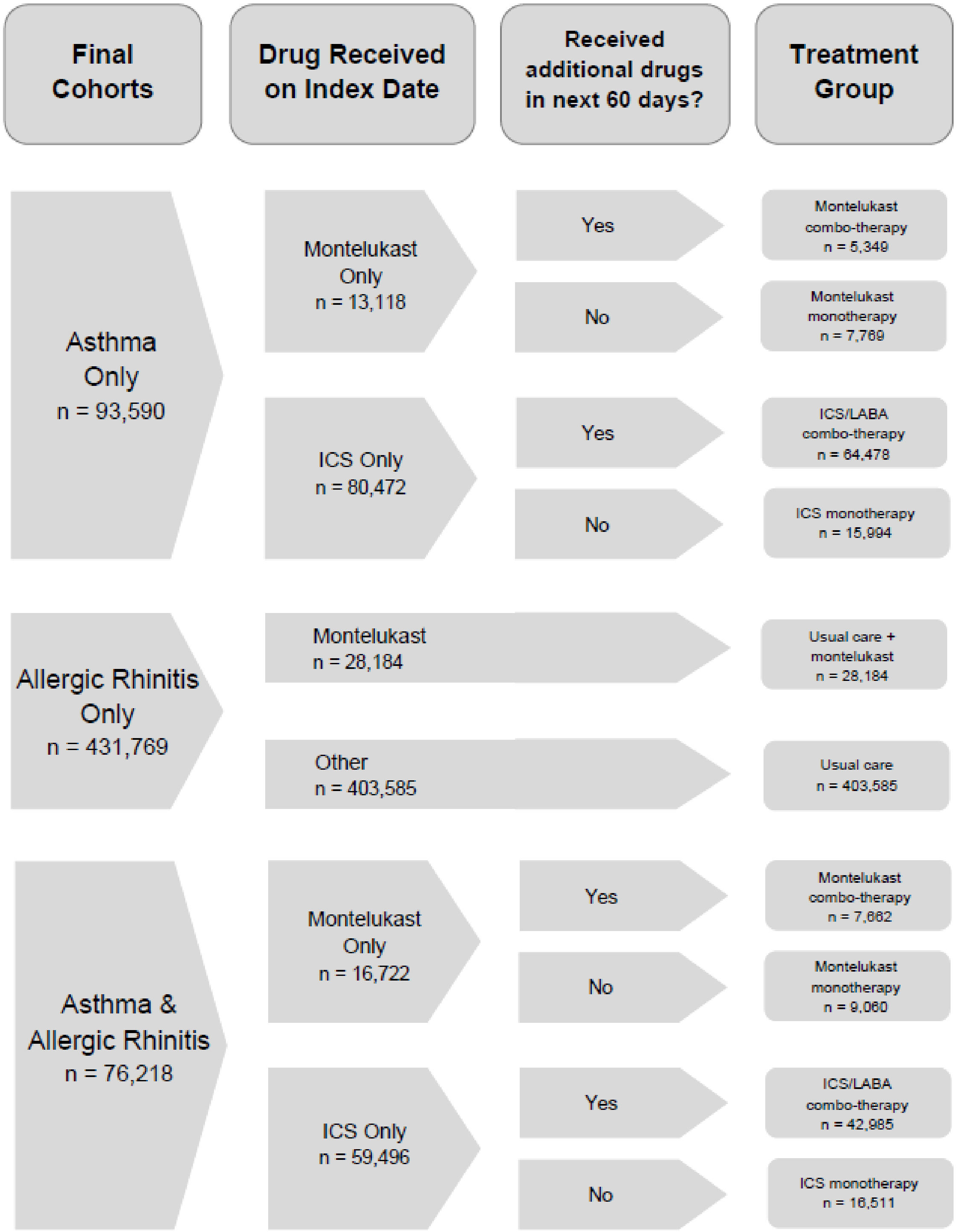

**Table 1.** Demographic and Clinical Characteristics of Study Patients

| Characteristic | Asthma Only | | | | Allergic Rhinitis Only | |
|---|---|---|---|---|---|---|
| | Montelukast Monotherapy | ICS Monotherapy | Montelukast Combo. | ICS Combination | Usual Care | Montelukast + Usual Care |
| | n = 7769 | n = 15 994 | n = 5349 | n = 64 478 | n = 403 585 | n = 28 184 |
| Age at Index, Mean/ SD | 51/ 16.5 | 49/ 16.7 | 47/ 16.3 | 49/ 16.3 | 49/ 16.9 | 48/ 16.1 |
| **Gender** | | | | | | |
| Male | 1859 (23.9) | 4492 (28.1) | 1345 (25.1) | 19 386 (30.1)* | 137 498 (34.1) | 9048 (32.1)* |
| Female | 5910 (76.1) | 11 502 (71.9) | 4004 (74.9) | 45 092 (69.9)* | 266 087 (65.9) | 19 136 (67.9)* |
| **Race** | | | | | | |
| White | 6617 (85.2) | 13 430 (84)* | 4110 (76.8) | 52 162 (80.9)* | 316 246 (78.4) | 23 638 (83.9)* |
| Black | 775 (10.0) | 1639 (10.2) | 799 (14.9) | 7904 (12.3)* | 48 785 (12.1) | 2794 (9.9)* |
| Asian | 132 (1.7) | 318 (2.0) | 123 (2.3) | 1381 (2.1) | 14 685 (3.6) | 635 (2.3)* |
| Hispanic | 118 (1.5) | 269 (1.7) | 156 (2.9) | 1629 (2.5)* | 13 779 (3.4) | 536 (1.9)* |
| Other | 127 (1.6) | 338 (2.1)* | 161 (3.0) | 1402 (2.2)* | 10 090 (2.5) | 581 (2.1)* |
| **BMI category** | | | | | | |
| < 25 | 805 (10.4) | 2390 (14.9)* | 635 (11.9) | 7403 (11.5) | 75 380 (18.7) | 4814 (17.1)* |
| 25 ≤ 30 | 1078 (13.9) | 3076 (19.2)* | 869 (16.2) | 10 355 (16.1) | 93 249 (23.1) | 5904 (20.9)* |
| 30+ | 2069 (26.6) | 5468 (34.2)* | 1768 (33.1) | 22 343 (34.7)* | 120 185 (29.8) | 8093 (28.7)* |
| N/A | 3817 (49.1) | 5060 (31.6)* | 2077 (38.8) | 24 377 (37.8) | 114 771 (28.4) | 9373 (33.3)* |
| **Smoking status** | | | | | | |
| Current | 296 (3.8) | 1152 (7.2)* | 381 (7.1) | 5673 (8.8)* | 28 158 (7.0) | 1566 (5.6)* |
| Former | 500 (6.4) | 1540 (9.6)* | 474 (8.9) | 6370 (9.9)* | 36 802 (9.1) | 2358 (8.4)* |
| Never | 1349 (17.4) | 3544 (22.2)* | 1241 (23.2) | 13 585 (21.1)* | 104 309 (25.8) | 7466 (26.5)* |
| N/A | 5624 (72.4) | 9758 (61.0)* | 3253 (60.8) | 38 850 (60.3) | 234 316 (58.1) | 16 794 (59.6)* |
| **Comorbidity** | | | | | | |
| Hypertension | 1746 (22.5) | 4548 (28.4)* | 1131 (21.1) | 16 701 (25.9)* | 121 897 (30.2) | 6991 (24.8)* |
| Sleep disorder | 650 (8.4) | 2006 (12.5)* | 476 (8.9) | 6781 (10.5)* | 43 416 (10.8) | 2824 (10.0)* |
| Sinusitis | 475 (6.1) | 1529 (9.6)* | 447 (8.4) | 4489 (7.0)* | 42 366 (10.5) | 3951 (14.0)* |
| Tonsillitis | 7 (0.1) | 35 (0.2) | 19 (0.4) | 163 (0.3) | 1340 (0.3) | 96 (0.3) |
| Rhinorrhea | - | - | - | - | 9 (0.0) | 2 (0.0) |
| Acute Upper Respiratory Infection | 281 (3.6) | 942 (5.9)* | 297 (5.6) | 3547 (5.5) | 25 455 (6.3) | 1837 (6.5) |
| Conjunctivitis | 47 (0.6) | 191 (1.2)* | 61 (1.1) | 610 (0.9) | 6222 (1.5) | 640 (2.3)* |
| Chronic Otitis Media | 31 (0.4) | 143 (0.9) | 28 (0.5) | 358 (0.6) | 3146 (0.8) | 225 (0.8) |
| Pharyngitis | 176 (2.3) | 614 (3.8)* | 182 (3.4) | 1958 (3.0) | 16 582 (4.1) | 1139 (4.0) |
| Nasal polyposis | 19 (0.2) | 66 (0.4) | 13 (0.2) | 156 (0.2) | 956 (0.2) | 163 (0.6)* |

* Indicates a significant difference (p<0.05) between treatment changes containing montelukast vs. not.

**Table 1.** Demographic and Clinical Characteristics of Study Patients (continued)

| Characteristic | Asthma and Allergic Rhinitis | | | | | | | |
|---|---|---|---|---|---|---|---|---|
| | Montelukast Monotherapy | | ICS Monotherapy | | Montelukast Combination | | ICS Combination | |
| | n = 9060 | | n = 16 511 | | n = 7662 | | n = 42 985 | |
| Age at Index, Mean/SD | 48/ | 15.8 | 47/ | 15.8 | 44/ | 15.5 | 47/ | 15.7 |
| **Gender** | | | | | | | | |
| Male | 2257 | (24.9) | 4326 | (26.2)* | 1854 | (24.2) | 11 936 | (27.8)* |
| Female | 6803 | (75.1) | 12 185 | (73.8)* | 5808 | (75.8) | 31 049 | (72.2)* |
| **Race** | | | | | | | | |
| White | 7695 | (84.9) | 13 522 | (81.9)* | 6075 | (79.3) | 34 764 | (80.9)* |
| Black | 894 | (9.9) | 1785 | (10.8)* | 948 | (12.4) | 5138 | (12.0) |
| Asian | 164 | (1.8) | 447 | (2.7)* | 184 | (2.4) | 910 | (2.1) |
| Hispanic | 170 | (1.9) | 384 | (2.3)* | 238 | (3.1) | 1195 | (2.8) |
| Other | 137 | (1.5) | 373 | (2.3)* | 217 | (2.8) | 978 | (2.3)* |
| **BMI category** | | | | | | | | |
| < 25 | 1191 | (13.1) | 2653 | (16.1)* | 1145 | (14.9) | 5355 | (12.5)* |
| 25 ≤ 30 | 1436 | (15.8) | 3167 | (19.2)* | 1379 | (18.0) | 7264 | (16.9)* |
| 30+ | 2501 | (27.6) | 5283 | (32)* | 2337 | (30.5) | 13 936 | (32.4)* |
| N/A | 3932 | (43.4) | 5408 | (32.8)* | 2801 | (36.6) | 16 430 | (38.2)* |
| **Smoking status** | | | | | | | | |
| Current | 345 | (3.8) | 893 | (5.4)* | 428 | (5.6) | 2759 | (6.4)* |
| Former | 571 | (6.3) | 1457 | (8.8)* | 658 | (8.6) | 3922 | (9.1) |
| Never | 1950 | (21.5) | 3998 | (24.2)* | 2088 | (27.3) | 10 266 | (23.9)* |
| N/A | 6194 | (68.4) | 10 163 | (61.6)* | 4488 | (58.6) | 26 038 | (60.6)* |
| **Comorbidity** | | | | | | | | |
| Hypertension | 1848 | (20.4) | 4069 | (24.6)* | 1418 | (18.5) | 9744 | (22.7)* |
| Sleep disorder | 865 | (9.5) | 1864 | (11.3)* | 713 | (9.3) | 4569 | (10.6)* |
| Sinusitis | 1004 | (11.1) | 2177 | (13.2)* | 1034 | (13.5) | 4785 | (11.1)* |
| Tonsillitis | 24 | (0.3) | 58 | (0.4) | 31 | (0.4) | 94 | (0.2)* |
| Rhinorrhea | - | | - | | - | | - | |
| Acute Upper Respiratory Infection | 447 | (4.9) | 1224 | (7.4)* | 556 | (7.3) | 2729 | (6.3)* |
| Conjunctivitis | 202 | (2.2) | 405 | (2.5) | 251 | (3.3) | 1083 | (2.5)* |
| Chronic Otitis Media | 62 | (0.7) | 169 | (1.0) | 65 | (0.8) | 315 | (0.7) |
| Pharyngitis | 296 | (3.3) | 751 | (4.5)* | 343 | (4.5) | 1551 | (3.6)* |
| Nasal polyposis | 46 | (0.5) | 86 | (0.5) | 60 | (0.8) | 268 | (0.6) |

* Indicates a significant difference (p<0.05) between treatment changes containing montelukast vs. not

**Table 2.** Number (and Percentage) of Patients with a Change in Initial Treatment Regimen during the Follow-Up Period

| Treatment Pattern, n (%) | Asthma Only | | | | Asthma and Allergic Rhinitis | | | |
|---|---|---|---|---|---|---|---|---|
| | Montelukast Monotherapy | ICS Monotherapy | Montelukast Combination | ICS Combination | Montelukast Monotherapy | ICS Monotherapy | Montelukast Combination | ICS Combination |
| **Asian** | | | | | | | | |
| | n = 132 | n = 318 | n = 123 | n = 1,381 | n = 164 | n = 447 | n = 184 | n = 910 |
| Switch to Another Medication | 4 (3.0) | 13 (4.1) | 1 (0.8) | 12 (0.9) | 3 (1.8) | 12 (2.7) | 2 (1.1) | 13 (1.4) |
| Augment with Another Medication | 35 (26.5) | 50 (15.7)* | 71 (57.7) | 763 (55.2) | 47 (28.7) | 89 (19.9)* | 86 (46.7) | 496 (54.5)* |
| No Additional Medication | 93 (70.5) | 255 (80.2)* | 51 (41.5) | 606 (43.9) | 114 (69.5) | 346 (77.4)* | 96 (52.2) | 401 (44.1)* |
| **Non Asian** | | | | | | | | |
| | n = 7637 | n = 15 676 | n = 5226 | n = 63 097 | n = 8896 | n = 16 064 | n = 7478 | n = 42 075 |
| Switch to Another Medication | 172 (2.3) | 564 (3.6)* | 55 (1.1) | 857 (1.4) | 253 (2.8) | 673 (4.2)* | 91 (1.2) | 677 (1.6)* |
| Augment with Another Medication | 2106 (27.6) | 2701 (17.2)* | 2706 (51.8) | 33 622 (53.3) | 2589 (29.1) | 3190 (19.9)* | 3592 (48.0) | 23 783 (56.5)* |
| No Additional Medication | 5359 (70.2) | 12 411 (79.2)* | 2465 (47.2) | 28 618 (45.4) | 6054 (68.1) | 12 201 (76.0)* | 3795 (50.7) | 17 615 (41.9)* |

*Indicates a significant difference ($p<0.05$) between treatment changes containing montelukast vs. not

In the follow-up period, short-acting beta-agonist (SABA) rescue inhalers were the most widely prescribed additional medication (Table 3). Among Asians with asthma only, a larger proportion of patients on Montelukast monotherapy received SABAs than the patients on ICS monotherapy (25% compared to 14%). This difference was smaller for non-Asian patients – 21% compared to 19%. Differences for other therapies were small and statistically insignificant. Among patients with a diagnosis of asthma and AR, differences between monotherapies and combination therapies were not statistically significant for Asian patients. However, differences were significant for non-Asians, though slight – 23% for montelukast monotherapy vs. 21% for ICS monotherapy; 29% for montelukast combination therapy vs. 27% for ICS combination therapy. Immunotherapy, cromolyn, and theophylline are rarely prescribed in the follow up period for any diagnosis, treatment, or race group.

**Utilization of Physician Services**

Differences in mean number of all-cause office visits were noted for Asians, as well as non-Asians (Table 4), when adjusted for patient demographic and clinical characteristics (age, gender, BMI, smoking status, and presence of asthma-related comorbidities). Among Asians with asthma only, the mean number of visits was higher for montelukast therapy than ICS therapy – for monotherapy it was 7.4 visits for montelukast vs. 4.3 visits for ICS; for combination therapy it was 6.7 visits for montelukast vs. 5.0 visits for ICS. There were no differences for non-Asian patients. For asthma & AR patients, both Asians and non-Asians using montelukast monotherapy had fewer visits than ICS monotherapy (Asians - 3.6 vs. 4.7; Non-Asians – 5.4 vs. 5.6). For the same disease cohort, Asian patients using montelukast combination therapy had a slightly lower rate (though not statistically significant) than those using ICS combination therapy. This result was reversed for non-Asian patients. AR patients receiving usual care with montelukast had lower number of visits than those without montelukast for both Asians and non-Asians.

Spirometry testing was not widely ordered among the patients, affecting between 1-8% of Asians and 1-9% of non-Asians, and varying by cohort and treatment condition (Table 5). Among Asians it was more likely to be ordered for ICS combination versus montelukast combination treatments of Asthma & AR. Among non-Asians spirometry orders occurred relatively more often for ICS combination therapy than montelukast combination therapy for the asthma-only cohort, more often for usual care for AR with montelukast than without.

Prescriptions for oral corticosteroids were noted for 5-20% of Asians and 10-25% of non-Asians. Among Asians, a greater number of patients on both ICS monotherapy and combination therapy were prescribed oral corticosteroids than comparable montelukast therapies, though the differences are not statistically significant. The exception was for AR-Only patients, where 12% of montelukast patients received a prescription compared to 5% of those on usual care. A similar pattern was observed among non-Asian AR-Only patients, where a significantly larger proportion of patients on usual care plus montelukast had a prescription compared to those without montelukast (14.1% vs. 10.4%).

**Table 3.** Number (and Percentage) of Patients Receiving Particular Medications in the Follow-Up Period

| Medication, n(%) | Asthma Only: Montelukast Monotherapy | Asthma Only: ICS Monotherapy | Asthma Only: Montelukast Combination | Asthma Only: ICS Combination | Allergic Rhinitis Only: Usual Care | Allergic Rhinitis Only: Montelukast + Usual Care |
|---|---|---|---|---|---|---|
| **Asian** | | | | | | |
| | n = 132 | n = 318 | n = 123 | n = 1381 | n = 14 685 | n = 635 |
| Inhaled Corticosteroids (ICS) | 11 (8.3) | 58 (18.2)* | 33 (26.8) | 161 (11.7)* | | |
| Long-Acting Beta-Agonists (LABAs) | - | - | 2 (1.6) | 5 (0.4) | | |
| ICS+LABA | 15 (11.4) | 21 (6.6) | 26 (21.1) | 242 (17.5) | | |
| Leukotriene Modifiers | 13 (9.8) | 9 (2.8)* | 12 (9.8) | 84 (6.1) | | |
| Immunomodulators | - | - | - | - | | |
| Short-Acting Beta-Agonists (SABAs) | 33 (25.0) | 45 (14.2)* | 36 (29.3) | 354 (25.6) | | |
| Cromolyn and Theophylline | - | - | 3 (2.4) | 7 (0.5) | | |
| Antihistamines | | | | | 3312 (22.6) | 115 (18.1)* |
| Nasal Corticosteroids | | | | | 2391 (16.3) | 115 (18.1) |
| Other AR medication | | | | | 505 (3.4) | 6 (0.9)* |
| **Non Asian** | | | | | | |
| | n = 7637 | n = 15 676 | n = 5226 | n = 63 097 | n = 388 900 | n = 27 549 |
| Inhaled Corticosteroids (ICS) | 660 (8.6) | 2390 (15.2)* | 1426 (27.3) | 5987 (9.5)* | | |
| Long-Acting Beta-Agonists (LABAs) | 25 (0.3) | 58 (0.4) | 56 (1.1) | 243 (0.4)* | | |
| ICS+LABA | 821 (10.8) | 805 (5.1)* | 1275 (24.4) | 9852 (15.6)* | | |
| Leukotriene Modifiers | 472 (6.2) | 473 (3.0)* | 410 (7.8) | 4070 (6.5)* | | |
| Immunomodulators | 5 (0.1) | 1 (0.0) | 7 (0.1) | 31 (0.0) | | |
| Short-Acting Beta-Agonists (SABAs) | 1623 (21.3) | 3007 (19.2)* | 1570 (30.0) | 15 237 (24.1)* | | |
| Cromolyn and Theophylline | 20 (0.3) | 19 (0.1)* | 32 (0.6) | 224 (0.4)* | | |
| Antihistamines | | | | | 55 647 (14.3) | 4236 (15.4)* |
| Nasal Corticosteroids | | | | | 53 214 (13.7) | 4493 (16.3)* |
| Other AR medication | | | | | 6296 (1.6) | 417 (1.5) |

*Indicates a significant difference ($p<0.05$) between treatment regimens containing montelukast vs. not

**Table 3.** Number (and Percentage) of Patients Receiving Particular Medications in the Follow-Up Period (continued)

| Medication, n(%) | Asthma and Allergic Rhinitis: Montelukast Monotherapy | ICS Monotherapy | Montelukast Combination | ICS Combination |
|---|---|---|---|---|
| **Asian** | | | | |
| | n = 164 | n = 447 | n = 184 | n = 910 |
| Inhaled Corticosteroids (ICS) | 19 (11.6) | 79 (17.7) | 48 (26.1) | 152 (16.7)* |
| Long-Acting Beta-Agonists (LABAs) | - | 2 (0.4) | 1 (0.5) | 3 (0.3) |
| ICS+LABA | 20 (12.2) | 36 (8.1) | 35 (19.0) | 171 (18.8) |
| Leukotriene Modifiers | 16 (9.8) | 17 (3.8)* | 18 (9.8) | 115 (12.6) |
| Immunomodulators | - | - | - | - |
| Short-Acting Beta-Agonists (SABAs) | 35 (21.3) | 88 (19.7) | 50 (27.2) | 208 (22.9) |
| Cromolyn and Theophylline | - | 1 (0.2) | 3 (1.6) | 11 (1.2) |
| Antihistamines | | | | |
| Nasal Corticosteroids | | | | |
| Other AR medication | | | | |
| **Non Asian** | | | | |
| | n = 8896 | n = 16 064 | n = 7478 | n = 42 075 |
| Inhaled Corticosteroids (ICS) | 1131 (12.7) | 2781 (17.3)* | 2389 (31.9) | 6209 (14.8)* |
| Long-Acting Beta-Agonists (LABAs) | 29 (0.3) | 38 (0.2) | 54 (0.7) | 164 (0.4)* |
| ICS+LABA | 880 (9.9) | 1043 (6.5)* | 1625 (21.7) | 7862 (18.7)* |
| Leukotriene Modifiers | 712 (8.0) | 976 (6.1)* | 758 (10.1) | 4483 (10.7) |
| Immunomodulators | 5 (0.1) | 1 (0.0) | 4 (0.1) | 57 (0.1) |
| Short-Acting Beta-Agonists (SABAs) | 2026 (22.8) | 3410 (21.2)* | 2150 (28.8) | 11293 (26.8)* |
| Cromolyn and Theophylline | 20 (0.2) | 24 (0.1) | 50 (0.7) | 174 (0.4)* |
| Antihistamines | 1418 (15.9) | 2487 (15.5) | 1507 (20.2) | 7110 (16.9)* |
| Nasal Corticosteroids | 1736 (19.5) | 3679 (22.9)* | 2702 (36.1) | 9452 (22.5)* |
| Other AR medication | 154 (1.7) | 308 (1.9) | 255 (3.4) | 962 (2.3)* |

*Indicates a significant difference ($p<0.05$) between treatment regimens containing montelukast vs. not

**Table 4.** Unadjusted and Adjusted** Mean Number of Physician Office Visits (Any Reason and Asthma/AR Related) during the Follow-Up Period

| | Asthma Only | | | | Allergic Rhinitis Only | |
|---|---|---|---|---|---|---|
| **Utilization Measure, (Mean/SD)** | **Montelukast Monotherapy** | **ICS Monotherapy** | **Montelukast Combination** | **ICS Combination** | **Usual Care** | **Montelukast + Usual Care** |
| **Asian** | | | | | | |
| | n = 132 | n = 318 | n = 123 | n = 1381 | n = 14 685 | n = 635 |
| **Office Visit, Any Reason:** | | | | | | |
| Unadjusted Mean | 4.3/ 5.5 | 4.5/ 4.4 | 5.5/ 5.4 | 5.0/ 4.9 | 4.1/ 3.8 | 3.6/ 3* |
| Adjusted Mean | 7.4/ 2.5 | 4.3/ 1.1* | 6.7/ 1.8 | 5.0/ 1.5* | 4.3/ 0.7 | 3.6/ 0.6* |
| **Non Asian** | | | | | | |
| | n = 7637 | n = 15 676 | n = 5226 | n = 63 097 | n = 388 900 | n = 27 549 |
| **Office Visit, Any Reason:** | | | | | | |
| Unadjusted Mean | 4.8/ 4.6 | 4.8/ 4.4 | 5.1/ 4.3 | 4.9/ 4.6* | 4.5/ 4.1 | 4.4/ 4.3* |
| Adjusted Mean | 5.3/ 0.9 | 5.3/ 0.9 | 5.4/ 0.9 | 5.4/ 0.9 | 4.8/ 0.8 | 4.6/ 0.8* |

| | Asthma and Allergic Rhinitis | | | |
|---|---|---|---|---|
| **Utilization Measure, (Mean/SD)** | **Montelukast Monotherapy** | **ICS Monotherapy** | **Montelukast Combination** | **ICS Combination** |
| **Asian** | | | | |
| | n = 164 | n = 447 | n = 184 | n = 910 |
| **Office Visit, Any Reason:** | | | | |
| Unadjusted Mean | 4.1/ 3.9 | 4.4/ 4.0 | 4.7/ 4.1 | 4.9/ 4.5 |
| Adjusted Mean | 3.6/ 1.1 | 4.7/ 1.3* | 5.2/ 1.2 | 5.4/ 1.6 |
| **Non Asian** | | | | |
| | n = 8896 | n = 16 064 | n = 7478 | n = 42 075 |
| **Office Visit, Any Reason:** | | | | |
| Unadjusted Mean | 5.0/ 4.7 | 5.0/ 4.9 | 5.5/ 5.4 | 5.2/ 5.0* |
| Adjusted Mean | 5.4/ 1.1 | 5.6/ 1.1* | 5.9/ 1.2 | 5.7/ 1.2* |

* Indicates a significant difference ($p<0.05$) in utilization measures between treatments containing montelukast vs. not

** Adjusted estimates derived from covariate adjusted multivariate models (age, BMI, smoking status, asthma related comorbidities)

**Table 5.** Healthcare Utilization during the Follow-up Period

| Type of Utilization, n (%) | Asthma Only: Montelukast Mono. | Asthma Only: ICS Mono. | Asthma Only: Montelukast Combo. | Asthma Only: ICS Combo. | Allergic Rhinitis Only: Usual Care | Allergic Rhinitis Only: Montelukast + Usual Care |
|---|---|---|---|---|---|---|
| **Asian** | | | | | | |
| | n = 132 | n = 318 | n = 123 | n = 1381 | n = 14 685 | n = 635 |
| Orders for Spirometry Testing | 8 (6.1) | 16 (5.0) | 7 (5.7) | 72 (5.2) | 115 (0.8) | 14 (2.2)* |
| Orders for Education and Training for Patient Self Management | - | - | - | 3 (0.2) | 12 (0.1) | - |
| Prescriptons for any Oral Cortico-steroid | 16 (12.1) | 40 (12.6) | 25 (20.3) | 270 (19.6) | 800 (5.4) | 76 (12.0)* |
| **Non Asian** | | | | | | |
| | n = 7637 | n = 15 676 | n = 5226 | n = 63 097 | n = 388,900 | n = 27 549 |
| Orders for spirometry testing | 287 (3.8) | 677 (4.3)* | 308 (5.9) | 3645 (5.8) | 4236 (1.1) | 500 (1.8)* |
| Orders for Education and Training for Patient Self Management | 1 (0.0) | 5 (0.0) | 2 (0.0) | 25 (0.0) | 159 (0.0) | 11 (0.0) |
| Prescriptons for any Oral Cortico-steroid | 1323 (17.3) | 2443 (15.6)* | 1280 (24.5) | 13 712 (21.7)* | 40 429 (10.4) | 3874 (14.1)* |

| Type of Utilization, n (%) | Asthma and Allergic Rhinitis: Montelukast Mono. | Asthma and Allergic Rhinitis: ICS Mono. | Asthma and Allergic Rhinitis: Montelukast Combo. | Asthma and Allergic Rhinitis: ICS Combo. |
|---|---|---|---|---|
| **Asian** | | | | |
| | n = 164 | n = 447 | n = 184 | n = 910 |
| Orders for Spirometry Testing | 11 (6.7) | 19 (4.3) | 6 (3.3) | 68 (7.5)* |
| Orders for Education and Training for Patient Self Management | - | - | - | - |
| Prescriptons for any Oral Cortico-steroid | 18 (11.0) | 45 (10.1) | 34 (18.5) | 184 (20.2) |
| **Non Asian** | | | | |
| | n = 8896 | n = 16 064 | n = 7478 | n = 42 075 |
| Orders for spirometry testing | 524 (5.9) | 968 (6.0) | 728 (9.7) | 3836 (9.1) |
| Orders for Education and Training for Patient Self Management | 3 (0.0) | 5 (0.0) | 4 (0.1) | 12 (0.0) |
| Prescriptons for any Oral Cortico-steroid | 1697 (19.1) | 2892 (18.0)* | 1980 (26.5) | 10 698 (25.4) |

* Indicates a significant difference (p<0.05) in utilization measures between treatments containing montelukast vs. not

## DISCUSSION

We have examined use of montelukast and patterns of ambulatory care among patients with asthma and/or allergic rhinitis in the United States. Our approach involved a retrospective analysis of electronic medical records (EMR) from a large ambulatory database representing broad geographic reach and wide variation in practice settings. As data are limited on the use of montelukast and management of these conditions specifically among Asian patients, we stratified all patients by race so as to allow contrasts between Asian and non-Asian patients.

Our findings suggest that use of montelukast is similar on an overall basis between Asian and non-Asian asthma patients (with or without AR), but the way in which the product is used differs, with Asians more likely to receive it as part of a combination therapy regimen. Reasons for this treatment pattern variation are unknown, but could reflect differences in the clinical presentation of Asian versus non-Asian patients or in the physicians who treat them.

In the year following treatment, we found that monotherapies for asthma-only patients were less likely to be modified than combination therapies. Montelukast monotherapy had a slightly higher augmentation rate than ICS monotherapy, and this pattern was similar for Asian and non-Asian patients. For the Asthma & AR patients, montelukast combination therapy had a significantly lower augmentation rate than ICS-combination therapy, while the result was reversed for the monotherapy comparison. The most widely prescribed medications in the follow up period were SABAs. Montelukast monotherapy and combination therapy are more likely to be prescribed SABAs than ICS therapies for non-Asians for all disease cohorts, whereas this result is significant only for the asthma-only patients for Asians. However, this analysis may be complicated by the fact that severity of asthma is not available for patients in this database.

In general, patients with AR (either alone or in combination with asthma) had lower number of physician office visits for montelukast therapy than ICS therapy for both Asians and non-Asians. Spirometry testing was more likely to be ordered for ICS combination versus montelukast combination treatments of Asthma & AR among Asians. Among non-Asians it occurred relatively more often for ICS than montelukast combination therapy (asthma-only), and more often for usual care for AR with montelukast than without. A larger proportion of Asthma-only and AR-only patients with montelukast had prescriptions for oral corticosteroids in the non-Asian cohort, while there were no statistically significant differences in the Asian asthma-only cohort. What is striking is that AR-only patients, in both Asian and non-Asian groups, had a greater number of oral corticosteroid prescriptions for montelukast+usual care than for usual care alone. This could potentially point to the possibility that patients receiving both montelukast and usual care are more severe patients with undiagnosed asthma aggravating their condition. This hypothesis is supported by the result that these patients have a greater number of spirometry tests ordered as well which could be due to suspected asthma.

The evidence for the value of montelukast in treating asthma is well established. It is widely regarded as a valuable addition to ICS in treating asthma,[14,15,21,22] although there is some evidence that on its own, montelukast may be less effective than ICS alone.[23] The evidence for montelukast's effectiveness in treating AR is more mixed. While a research review[24] cites numerous studies which disagree over the effectiveness of montelukast in treating AR, other work[25] concludes that the available clinical evidence supports the use of montelukast in combination with other medications (primarily antihistamines) as an effective AR treatment. These studies generally consider effectiveness in terms of physiological functioning or reduction of negative events such as exacerbations. When patient race is considered, studies typically include it as a factor in regression models and report no effect.[21,25,26] In contrast, our study has characterized utilization in terms of a more global measure, all-cause office visits, and stratified the analyses by race, allowing us to consider the prescribing, modification, and effectiveness profile of montelukast in Asian and non-Asian samples. In doing so, we have found that differences exist between Asian and non-Asian patients in terms of how montelukast is incorporated into the treatment plan and in the high level outcome that may be attributable in part to it.

While this is a large retrospective national study, there are several points that may affect its generalizability. Most importantly, as used in this study, "treatment" refers to prescribed treatment. The data offer no way of assessing patient compliance with treatment plans. The considerable variability in patient compliance[27] introduces a degree of uncertainty in assessing outcomes in that patient behavior after leaving the physician's office is a black box. This may be complicated to the extent that compliance is associated with psychosocial norms that may characterize ethnic or racial subgroups in the United States.

Attitudes towards health care are driven by cultural norms, a concern that cannot be addressed by the data available from the EMR. In light of the changing composition of the US population, a deeper investigation into differences between Asian and other racial and ethnic groups is warranted. In identifying patients as Asian, the EMR does not make a distinction between people from the many cultures of Asia, including Japan, China, Korea, Southeast Asia, Indonesia, and the Indian subcontinent. Similarly, a classification of non-Asian does not distinguish between Caucasians, African-Americans, Middle-Easterners, or Latinos. An extension of the current study, facilitated by the acquisition of supplemental data, will address this concern by allowing a more precise segmentation of the patient population by race.

A related issue is that, the EMR contains no data on whether patients are US born, naturalized citizens, or immigrants, which might act as a proxy for how integrated into US culture they are. Patients from non-Western cultures may have attitudes, ideas and values about health, illness, and treatment that differ significantly from the US mainstream. Their values and expectations about patient and service provider roles will affect how services are used and the degree to which patients are cooperative and compliant with treatment.[28] For example, more fine-grained comparisons might segment asthma patients' national origin as Chinese, South Asian, Latino, African American, and Caucasian, a level of detail that is generally unavailable in EMR and claims records.

Also unaddressed in this study, again due to unavailable data, is the severity of asthma. Additional information on this dimension of patient health would allow a more precise assessment of the need for health services. Similarly, the availability of other ambulatory measures (e.g., reduction in symptoms, increase in patient-reported comfort, and functional assessments of daily living) which are not systematically available, would be valuable adjuncts in assessing the real-world impact of montelukast on patient health.

## CONCLUSIONS

Using ambulatory care data, this study has demonstrated that there are real world differences in the treatment of asthma and allergic rhinitis among Asian and non-Asian patients. Overall, the use of montelukast was similar between Asian and non-Asian asthma patients (with or without AR), but Asians were more likely to receive it as part of a combination therapy regimen. Asian patients with both asthma and AR were found to have lower service utilization rates (medication augmentation rate, number of physician office visits, orders for spirometry testing) if their therapy included montelukast, whereas for non-Asians there was no significant difference between regimens with or without montelukast. Future research should explore the reasons for these differences and whether they can be replicated in non-US settings.

## Declaration of Competing Interests

This study was sponsored by Merck, Kenilworth, NJ. Dr. Vasey, Dr. Huang, Mr. Wang and Dr. Thompson declare that they have no competing interests. Dr. Bagga is an employee of the sponsor (Merck, Kenilworth, NJ).

## Acknowledgements

Joseph Vasey (JV), Shalini Bagga (SB), Huan Huang (HH) and David Thompson (DT) designed the research methods. JV, HH, Tongsheng Wang (TW) and DT collected and analyzed the data. All authors contributed to data interpretation, made substantive contributions to the manuscript, and had final approval of the article.
